# Pernicious Effects of Alternating Current of High Voltage1Read at the eighty-fourth semiannual meeting of the Medical Society of the County of Erie, June 13,1905.

**Published:** 1905-07

**Authors:** Frederick H. Millener

**Affiliations:** Buffalo, N. Y.


### Pernicious Effects of Alternating Current of High Voltage.[1]

By FREDERICK H. MILLENER, M. D., Buffalo, N. Y.

THE Niagara frontier has become the great center of mechanical electrical development, and is destined to be at the head in that regard for many years to come, so, naturally, one would expect in this field, that is pregnant with all the possibilities of electricity, there would first be observed such phenomena as result from the immense quantities of this vital force produced here. Just as in the mining regions one looks for conditions arising from the occupation of mining.

In this brief paper I wish to present one phase of the great subject of electricity and its relation to mankind, having reference specially to its pathological effect upon the health and lives of men engaged directly in the manufacture, transforming or transmission of this mysterious field. The Röntgen ray was a chance discovery, and so are many of the discoveries which are afterwards taken up by our medical profession and utilised for the benefit of humanity.

---

1. Read at the eighty-fourth semiannual meeting of the Medical Society of the County of Erie, June 13, 1905.

Some of the members of this society could bear witness to the fact that I have probably wasted much time dabbling in the various manifestations of electricity; but I wish to add that one result of the vast amount of time I have given to this subject has been the formation of a habit of watching and investigating everything pertaining to electricity that has come to my attention, and the pernicious influence of alternating currents of high voltage has engaged my earnest consideration.

I have made a careful investigation of eighteen cases during the past few months, and the phenomena observed have been so remarkable, and at the same time so uniform and unvarying, that I felt it to be my duty to present the results of my research to this society, as a problem, in the hope that in a multitude of councilors, there might be found the wisdom which would work out the remedy. In the cases which I shall present to your consideration, I have, for personal and prudential reasons which any professional man will readily understand, omitted the names of the persons who have detailed to me the symptoms and the conditions which have developed in their several individual cases. It would do no good to give their names to the public, and it might interfere in some way with the tenure of their employment, as great corporations, like the great power companies, do not usually relish having their employes publish conditions attending their employment which might deter others from engaging in the same work.

Briefly, however, I may state that all of the facts stated to me have been voluntary, and not the result of questioning, and I am sure that you will agree with me that the similarity of the symptoms points to but one conclusion (and that the one reached by me) that continuous employment in the immediate presence of electric generators or transformers, where one is continuously in an atmosphere heavily charged with electricity, or ozone, or some light or ray as yet undiscovered, results in such disturbed conditions of the digestive fluids or of the secretions of the stomach and its cognate glands and organs, as to greatly impair the digestive function; and that persons so employed lose their appetite, and become of an almost chalky complexion, and experience pain and distress after partaking food, and often have to obtain short periods of vacation in order to recover their ability to eat and digest their foods in a normal manner.

Everybody has observed the exhilaration felt, not only by human beings, but by birds and animals and even by insects, just before an electrical storm. Children run and jump and ramble on the lawn or in the field, and feel almost as though they could fly; the young of animals frisk and sport about, birds dart through the air, and the insect is specially conspicuous in his tantalising capers. But when the storm has at last broken, and the lightening flashed, and the air smells of ozone and of other electric fluids, there is a feeling of depression, and everything alive seeks to find some restful place where it can keep very quiet. And when the storm had passed away, normal conditions return, and the air is said to have been cleared again.

This is the most common experience. Another common experience is to find, immediately after a thunder storm, that all the sweet milk in the house has turned sour in an incredibly short time. Here we can plainly see a chemical force has been at work. What is that force? It has been explained in a variety of ways. Take your choice of the explanations. I have my ideas.

I have visited the largest, in fact practically all, of the power houses and transforming plants in this entire Niagara frontier, notably those in Buffalo, Niagara Falls, Lockport, Niagara Falls, Ontario, and many of the smaller plants; but it is particularly of the larger plants I wish to speak, where the manufacture of electricity or its transformation is the sole business of the plant.

On Saturday night last, I visited one of the power plants and one of the men in charge told me that after working for some time in the plant he found that his tea and coffee did not agree with him. He drank no liquors of any kind. Then he began bringing a bottle of milk with him for his lunch, but he said that did not work, because the milk always turned sour before noon, although it was delivered fresh the same morning. To test this, I personally went to a nearby barn and milked a quart from an obliging cow, and brought it to the plant and placed it where it would have the direct influence of the waste electricity from one of the large transformers, and in less than 90 minutes the milk was sour,—the chemical effect of the high voltage alternating current. The man himself had been at work there for 6 months, and he said that when he came to the plant he was hearty, red faced, full blooded, had a good appetite, good digestion, and was a perfectly well man. Now his face and hands were almost as white as chalk, he had no appetite, and when he ate his lunch, he nearly always felt distress from it in a short time, if he remained in the plant. Whereas, if he went home to lunch and stayed away during the afternoon, he had no such distressing feelings. The same was true of breakfast or dinner. If he ate his lunch at home, and returned to the plant, he would feel the distress, just the same as if he had eaten it in the plant.

This condition was not due in any degree to a sedentary life, for his work kept him very active, and he got all the exercise a man ought to have to keep well and healthy. Neither was it due in any degree to bad ventilation, as in these power plants there is always the very best ventilation, windows and doors are open, and to test the currents of air in one of them, I carried a lighted candle about, and I found a uniform current of air from the outside inward towards the machines. These power plants are kept scrupulously clean; drainage, lighting, heating, and the like, are perfect. In fact no stoves or heating apparatus is ever employed, as the waste energy in the form of heat which comes from the machines is always found sufficient to keep the buildings warm enough winter or summer. One would say that a power house was an ideal place in which to work, so far as all conditions of hygiene or sanitation are concerned, and so it is; but there is that mysterious fluid flowing all around about you, above, below, and on every side. Is it ozone? Is it the *x*-ray? Is it the violet, or the ultra-violet ray? Or is it some ray or light which science has not yet observed? What is it that throws the digestive apparatus into such disorder?

CASE I.—Mr. P., age 29; married; was employed around transformers, transforming current of 23,000 volts, 11,000 volts, and 2,300 volts per year. After having been near the machines for six or seven months he began to experience abdominal pains, became constipated, lost color and became irritable. This man went to several physicians, who, after examining him, would prescribe a physic and tell him that there was nothing particular the matter with him. He found that the only relief he could get from his trouble was to take a vacation. He has had to seek other employment.

CASE II.—J. E. B., age 27; worked around transformers two years, suffered from indigestion, constipation, and pallor, lack of energy and irritableness, with a desire to go to stool, the results of which are negative, and when he has a passage they are ill-smelling but normal in color. As soon as he leaves the station for a couple of days he feels better.

CASE III.—B. W. H.; married; age 30; good habits; has worked around transformers for five years, has been in three different stations. Four months after first going to work he began to have pains across his abdomen, loss of appetite and extreme pallor. He was obliged to "lay off" from his work frequently. Physicians were inclined to believe he was coming down with tuberculosis.

I have a record of fifteen more cases, all of whose symptoms are similar, therefore, I will not read them, but any of the medical profession who are interested I would be pleased to place in communication with them. These men are all at the present time able to work, but their work is not the pleasure it should be.

I can add but little more. I have stated a problem to the physicians of Erie County. Will the honor belong to one of us of discovering the remedy? What that remedy will be I cannot say. It may be some sort of a grounding of this wasted fluid that now fills the rooms of the power houses. It may be some neutralising ray, or some form of insulation which can be applied. It may be any one of many things, but which one, none of us is at present prepared to state. It may be some great electrician like the immortal Edison who will find the remedy, instead of one of our profession. For when I had the pleasure of an intensely interesting visit with Mr. Edison in his laboratory last month, I found him as deeply absorbed with things new and marvelous as he ever was in the whole of his marvelous lifetime. I shall shortly submit this problem to him, for my own personal satisfaction, even if without the hope of any solution.

What does this mean to the men engaged in this business? First, it means personal discomfort and distress, which alone would make it enough of a problem to engage our serious attention. Then, again, it means that these men will be put in substandard lists for purposes of life insurance. It means, possibly, deterioration more or less permanent to the individual and to his children. It should be of sufficient interest to our profession to warrant further study and investigation. If my paper has the effect of awakening an interest in this problem, it will have served its purpose.

139 N. Pearl Street.

